# Deep Learning With Chest Radiographs for Making Prognoses in Patients With COVID-19: Retrospective Cohort Study

**DOI:** 10.2196/42717

**Published:** 2023-02-16

**Authors:** Hyun Woo Lee, Hyun Jun Yang, Hyungjin Kim, Ue-Hwan Kim, Dong Hyun Kim, Soon Ho Yoon, Soo-Youn Ham, Bo Da Nam, Kum Ju Chae, Dabee Lee, Jin Young Yoo, So Hyeon Bak, Jin Young Kim, Jin Hwan Kim, Ki Beom Kim, Jung Im Jung, Jae-Kwang Lim, Jong Eun Lee, Myung Jin Chung, Young Kyung Lee, Young Seon Kim, Sang Min Lee, Woocheol Kwon, Chang Min Park, Yun-Hyeon Kim, Yeon Joo Jeong, Kwang Nam Jin, Jin Mo Goo

**Affiliations:** 1 Division of Respiratory and Critical Care Department of Internal Medicine Seoul Metropolitan Government-Seoul National University Boramae Medical Center Seoul Republic of Korea; 2 College of Medicine Seoul National University Seoul Republic of Korea; 3 Department of Radiology Seoul National University Medical Research Center Seoul Republic of Korea; 4 AI Graduate School Gwangju Institute of Science and Technology Gwangju Republic of Korea; 5 Department of Radiology Seoul Metropolitan Government-Seoul National University Boramae Medical Center Seoul Republic of Korea; 6 Department of Radiology Kangbuk Samsung Hospital Seoul Republic of Korea; 7 Department of Radiology Soonchunhyang University Seoul Hospital Soonchunhyang University College of Medicine Seoul Republic of Korea; 8 Department of Radiology Research Institute of Clinical Medicine Jeonbuk National University-Biomedical Research Institute of Jeonbuk National University Hospital Jeonju Republic of Korea; 9 Department of Radiology Dankook University Hospital Cheonan Republic of Korea; 10 Department of Radiology Chungbuk National University Hospital Cheongju Republic of Korea; 11 Department of Radiology Kangwon National University Hospital Kangwon National University School of Medicine Chuncheon Republic of Korea; 12 Department of Radiology Keimyung University Dongsan Hospital Keimyung University School of Medicine Daegu Republic of Korea; 13 Department of Radiology Chungnam National University Hospital College of Medicine Daejeon Republic of Korea; 14 Department of Radiology Daegu Fatima Hospital Daegu Republic of Korea; 15 Department of Radiology Seoul St. Mary's Hospital College of Medicine, The Catholic University of Korea Seoul Republic of Korea; 16 Department of Radiology Kyungpook National University Hospital School of Medicine, Kyungpook National University Daegu Republic of Korea; 17 Department of Radiology Chonnam National University Hospital Gwangju Republic of Korea; 18 Department of Radiology Samsung Medical Center Sungkyunkwan University School of Medicine Seoul Republic of Korea; 19 Department of Radiology Seoul Medical Center Seoul Republic of Korea; 20 Department of Radiology Yeungnam University Hospital Yeungnam University College of Medicine Daegu Republic of Korea; 21 Department of Radiology and Research Institute of Radiology Asan Medical Center University of Ulsan College of Medicine Seoul Republic of Korea; 22 Department of Radiology Ewha Womans University Seoul Hospital Seoul Republic of Korea; 23 Department of Radiology Pusan National University Hospital Pusan National University School of Medicine and Biomedical Research Institute Busan Republic of Korea

**Keywords:** COVID-19, deep learning, artificial intelligence, radiography, thoracic, prognosis, AI model, prediction model, clinical outcome, medical imaging, machine learning

## Abstract

**Background:**

An artificial intelligence (AI) model using chest radiography (CXR) may provide good performance in making prognoses for COVID-19.

**Objective:**

We aimed to develop and validate a prediction model using CXR based on an AI model and clinical variables to predict clinical outcomes in patients with COVID-19.

**Methods:**

This retrospective longitudinal study included patients hospitalized for COVID-19 at multiple COVID-19 medical centers between February 2020 and October 2020. Patients at Boramae Medical Center were randomly classified into training, validation, and internal testing sets (at a ratio of 8:1:1, respectively). An AI model using initial CXR images as input, a logistic regression model using clinical information, and a combined model using the output of the AI model (as CXR score) and clinical information were developed and trained to predict hospital length of stay (LOS) ≤2 weeks, need for oxygen supplementation, and acute respiratory distress syndrome (ARDS). The models were externally validated in the Korean Imaging Cohort of COVID-19 data set for discrimination and calibration.

**Results:**

The AI model using CXR and the logistic regression model using clinical variables were suboptimal to predict hospital LOS ≤2 weeks or the need for oxygen supplementation but performed acceptably in the prediction of ARDS (AI model area under the curve [AUC] 0.782, 95% CI 0.720-0.845; logistic regression model AUC 0.878, 95% CI 0.838-0.919). The combined model performed better in predicting the need for oxygen supplementation (AUC 0.704, 95% CI 0.646-0.762) and ARDS (AUC 0.890, 95% CI 0.853-0.928) compared to the CXR score alone. Both the AI and combined models showed good calibration for predicting ARDS (*P*=.079 and *P*=.859).

**Conclusions:**

The combined prediction model, comprising the CXR score and clinical information, was externally validated as having acceptable performance in predicting severe illness and excellent performance in predicting ARDS in patients with COVID-19.

## Introduction

SARS-CoV-2 infection causes COVID-19 pneumonia of varying severity. As of March 18, 2022, the global cumulative number of confirmed COVID-19 cases was more than 464.8 million, with >6 million deaths [1]. The occurrence of new variants of SARS-CoV-2 makes appropriate medical resource allocation, based on COVID-19 severity, challenging. A reliable prediction model for COVID-19 pneumonia would help in screening patients at a high risk of progression to severe disease or respiratory failure in a timely manner [2]. While many COVID-19 prediction models have been suggested, most have not been sufficiently validated [3].

Chest radiography (CXR) is not recommended for confirmation of diagnosis or assessment of COVID-19 severity [4]. Most studies have developed prognosis prediction models using clinical information and chest computed tomography (CT) scans [5,6]. However, with the high volume of patients during the pandemic, CXR is used more widely than chest CT because of its rapid speed, better portability, and lower cost [7,8]. Additionally, the role of CXR has been reexamined using advanced deep learning (DL) techniques. A DL model could make prognoses for COVID-19 more accurately than conventional severity score systems [9]. CXR information can increase the accuracy of severity assessment or risk stratification. An artificial intelligence (AI) model using CXR alone performed well in predicting COVID-19 severity [10]. A recent study showed that an AI model with CXR had the potential to predict mortality more accurately [11]. An external validation study reported that predictive modeling with CXR and clinical information improved prognoses compared to clinical information alone or radiologist-derived severity scores [12].

We aimed to create an AI model using CXR and clinical variables to predict early recovery, severe illness, and acute respiratory distress syndrome (ARDS) in patients with COVID-19 and validate these models in an external cohort.

## Methods

Our study adhered to the TRIPOD (Transparent Reporting of a Multivariable Prediction Model for Individual Prognosis or Diagnosis) guidelines [13].

### Study Design and Eligibility Criteria

This retrospective longitudinal study included hospitalized patients with COVID-19 in an isolation ward who received negative pressure ventilation from February 2020 to October 2020. COVID-19 was diagnosed by confirmatory quantitative reverse transcription–polymerase chain reaction using upper or lower respiratory tract samples. A protocol was used for systematic questionnaires and anthropometric measurements to ascertain demographic information, initial symptoms, and comorbidities of the patients. Within 24 hours of hospitalization, the patients routinely underwent blood tests and anteroposterior-view CXR. Medical decisions regarding treatment and discharge to home were made by each physician based on guidance from the Korea Disease Control and Prevention Agency. We excluded patients with missing clinical information or when there were technical difficulties in reading CXR images due to compromised software compatibility.

For model training, validation, and internal testing, we used CXR images and clinical information from patients who were admitted at Boramae Medical Center (BMC), Seoul, Korea. For external testing, we used the Korean Imaging Cohort of COVID-19 (KICC-19) data set, which collects imaging data and clinical information from patients with COVID-19 at 17 medical centers in Korea. Details on the profiles of the KICC-19 data set were published in an earlier report [14].

### Variables and Measurements

Data were collected on baseline characteristics, including age, sex, BMI, smoking status, and comorbidities. A protocolized questionnaire was used at the BMC to identify the symptoms of patients with COVID-19, including abnormal smell or taste, myalgia, sore throat, cough, sputum, chest discomfort, dyspnea, fever or chills, rhinorrhea, and nausea or diarrhea. In the KICC-19 data set, we extracted information on symptoms such as cough, dyspnea, and fever. We obtained laboratory test results, including white blood cell counts and lymphocyte percentage, as well as C-reactive protein (CRP), procalcitonin, troponin-I, and lactate dehydrogenase levels. Information on treatment and disease severity was also acquired. Information was obtained on clinical outcomes, including hospital length of stay (LOS) and oxygen supplementation, as well as the use of a high-flow nasal cannula (HFNC), mechanical ventilator (MV), or extracorporeal membrane oxygenation (ECMO). ARDS was operationally defined as a medical condition needing an HFNC, MV, or ECMO.

We obtained 26,684 CXR images with information on the location and extent of pneumonia provided by the Radiological Society of North America (RSNA) pneumonia-detection challenge. We extracted the initial CXR images from the electronic medical records of patients hospitalized for COVID-19 at the BMC and registered them in the KICC-19.

### Study Outcomes

The primary outcome was the performance of the AI model in predicting clinical outcomes such as hospital LOS ≤2 weeks, need for oxygen supplementation, and development of ARDS based on CXR images. The output value from this model was defined as the CXR score. The secondary outcome was whether the performance of the prediction model using clinical information could be improved by combining it with the CXR score.

### Development of the AI Model

**Figure 1:**
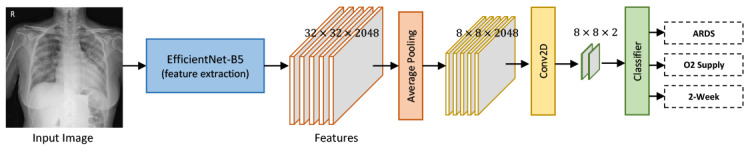
Illustration of the data flow model for artificial intelligence–assisted prediction. Our deep learning model was developed in two stages to ensure robust performance: (1) backbone training and (2) model training. ARDS: acute respiratory distress syndrome. Conv2D: Convolution 2D.

The DL model was implemented using the open-source PyTorch library (version 1.7.0+cu101) with the CUDA/cuDNN (versions 10.1 and 7.6.3, respectively) computing frameworks on a single graphics processing unit (Geforce RTX 3090; NVIDIA). The overall data flow and proposed model architecture is summarized in Figure 1. We developed our model in two stages, to ensure robust performance: (1) backbone training and (2) model training. First, we trained the backbone of our model with a data set (n=26,684) including information on the location and extent of pneumonia from the RSNA pneumonia-detection challenge to boost performance by learning robust features from a large quantity of data. The EfficientNet B5 architecture was used as the backbone architecture due to its computational efficiency and performance compared to those of other convolutional neural network architectures, such as ResNet and DenseNet. For backbone training, we attached a region proposal network, a region-of-interest pooling layer, and a classifier network to the backbone, which was pretrained on ImageNet, to configure the faster region-based convolutional neural network (RCNN) architecture (Multimedia Appendix 1, Figure S1). We trained the faster-RCNN model with the Adam optimizer under the following settings: learning rate of 1×10^–6^, learning decay rate of 0.8, learning rate decay step size of 4, and batch size of 3. We selected the model with the minimum validation loss, which was a combination of classification and bounding box regression loss. After the backbone training stage, the backbone learns to extract pertinent features for detecting pneumonia from posteroanterior or anteroposterior CXR images.

Next, we developed and trained the model for the classification of clinical outcomes. The backbone was followed by 3 branches: one each for hospital LOS ≤2 weeks, oxygen supplementation, and development of ARDS. Each branch consisted of an average pooling layer, a 2D convolution layer, a clinical data channel, and a fully connected layer. The outputs of the model were 3 probabilities between 0 and 1: one each for hospital LOS ≤2 weeks, oxygen supplementation, and development of ARDS; for each output, a probability >0.5 indicated a positive prediction. We initialized the weights of the models using the weights from the previous backbone training stage. We fixed the weights of the backbone during the model training stage to keep the feature extractor untouched. To train the proposed model, we used a configuration of the Adam optimizer with a learning rate of 1×10^–4^ and batch size of 12. We selected the model with minimum validation loss, that is, classification loss.

### Statistical Analyses

Demographic information, symptoms, laboratory test results, treatments, and study outcomes were compared among the training, validation, internal testing, and external testing sets using the Student 2-tailed *t* test or Mann-Whitney *U* test for continuous variables and the Pearson chi-square test or Fisher exact test for categorical variables. Univariate and multivariate logistic regression analyses were performed using the stepwise selection method. For 3 different prediction models for each clinical outcome (model 1, using the CXR score derived from the DL model; model 2, using clinical information derived from the multivariable regression model; and model 3, using both the CXR scores and clinical information from the multivariable regression model), performance was evaluated using sensitivity, specificity, positive predictive value (PPV), negative predictive value (NPV), accuracy, and the area under the receiver operating characteristic curve (AUROC). We considered an AUROC <0.7 suboptimal performance, 0.7 to 0.79 acceptable, 0.8 to 0.89 excellent, and ≥0.9 outstanding [15]. A comparison of the predictive performance between the 2 different models used the DeLong test [16] or the bootstrap test [17]. Statistical significance was set at *P*<.05. Calibration of the CXR score–based models (models 1 and 3) was evaluated by plotting the observed versus predicted probabilities and using the *P* value for the Spiegelhalter statistic [18,19]. Statistical significance in the Spiegelhalter *z* test indicates poor calibration. All the statistical analyses were performed using R (version 4.1.0; R Core Team).

### Ethics Approval

The Institutional Review Board Committee of the Boramae Medical Center (BMC) approved the study protocol and waived the need for informed consent for access to the electronic medical records (30-2020-307).

## Results

### Patient Characteristics

We used CXR images and clinical information from hospitalized patients with COVID-19 for model training (n=589), validation (n=75), and internal testing (n=75); we used patients with COVID-19 (n=467) registered in the KICC-19 for external testing. The median interval between symptom onset and CXR was 3 (IQR 1-6) days. The baseline characteristics of the combined total of 1206 patients are summarized in Table 1. The mean age was 53.4 years; 52.3% (n=631) were female; and 9.4% (n=113) were every-day smokers. Comorbidities included hypertension (n=317, 26.3%), diabetes mellitus (n=188, 15.6%), cancer (n=74, 6.1%), cardiovascular disease (n=73, 6.1%), chronic lung disease (n=50, 4.1%), chronic liver disease (n=26, 2.2%), and chronic kidney disease (n=20, 1.7%). The baseline characteristics of the patients in the training, validation, and internal testing sets are described in Multimedia Appendix 1, Table S1.

The clinical features of the 1206 patients are presented in Table 2. Cough, fever, and dyspnea were present in 53% (n=639), 51.7% (n=624), and 17.7% (n=213) of patients, respectively. At baseline assessment, mean white blood cell (WBC) count was 5024 cells/µL, with 29.4% lymphocytes. The median CRP and procalcitonin levels were 0.42 mg/dL and 0.03 ng/mL, respectively. Treatment for COVID-19 was remdesivir in 5.8% (n=70) and corticosteroids in 9.5% (n=115) of patients. Eligible patients were hospitalized for a median of 15 (IQR 11-24) days. HFNC, MV, and ECMO were used in 5.4% (n=65), 5% (n=60), and 1.5% (n=18) of patients, respectively. The clinical features of patients included in the training, validation, and internal testing sets are described in Multimedia Appendix 1, Table S2.

**Table 1:** Baseline characteristics of the patients diagnosed with COVID-19 in different data sets. *P* values were estimated using the Student 2-tailed *t* test or the Mann-Whitney *U* test for continuous variables and the Pearson chi-square test or Fisher exact test for categorical variables.

Characteristics			Total (n=1206)		Boramae Medical Center (n=739)	Korean Imaging Cohort of COVID-19 (n=467)		*P* value
Age, mean (SD)			53.4 (18.4)		54.5 (17.6)	51.8 (19.4)		.01
Female, n (%)			631 (52.3)		376 (50.9)	255 (54.6)		.23
Every-day smoker, n (%)			113 (9.4)		84 (11.3)	29 (6.2)		.004
**Comorbidities, n (%)**								
	Hypertension	317 (26.3)		213 (28.8)		104 (22.3)	.01	
	Diabetes mellitus	188 (15.6)		118 (16.0)		70 (15)	.71	
	Cancer	74 (6.1)		51 (6.9)		23 (4.9)	.20	
	Cardiovascular disease	73 (6.1)		50 (6.8)		23 (4.9)	.24	
	Chronic lung disease	50 (4.1)		38 (5.1)		12 (2.6)	.04	
	Chronic liver disease	26 (2.2)		24 (3.2)		2 (0.4)	.002	
	Chronic kidney disease	20 (1.7)		13 (1.8)		7 (1.5)	.91	

**Table 2:** Clinical manifestations of the patients diagnosed with COVID-19 in different data sets. *P* values were estimated using the Student 2-tailed *t* test or the Mann-Whitney *U* test for continuous variables and the Pearson chi-square test or Fisher exact test for categorical variables.

Clinical manifestations		Total (n=1206)	Boramae Medical Center (n=739)	Korean Imaging Cohort of COVID-19 (n=467)	*P* value
**Symptoms, n (%)**					
	Cough	639 (53)	417 (56.4)	222 (47.5)	.003
	Fever	624 (51.7)	376 (50.9)	248 (53.1)	.49
	Dyspnea	213 (17.7)	128 (17.3)	85 (18.2)	.79
**Laboratory tests**					
	White blood cells (cells/µL), mean (SD)	5024 (1941)	4984 (1932)	6007 (1926)	.005
	Lymphocytes (%), mean (SD)	29.4 (10.9)	29.5 (11.4)	29.2 (9.0)	.70
	C-reactive protein (mg/dL), median (IQR)	0.42 (0.12 to 1.83)	0.65 (0.17 to 3.14)	0.30 (0.10 to 0.38)	<.001
	Procalcitonin (ng/mL), median (IQR)	0.03 (0.01 to –0.04)	0.03 (0.02 to –0.05)	0.02 (0.00 to –0.04)	.02
**Treatment, n (%)**					
	Remdesivir	70 (5.8)	58 (7.8)	12 (2.6)	<.001
	Corticosteroid	115 (9.5)	74 (10)	41 (8.8)	.55
Length of stay (days), median (IQR)		15 (11 to 24)	13 (10 to 19)	22 (15 to 30)	<.001
Length of stay ≤2 weeks, n (%)		562 (46.6)	451 (61)	111 (23.8)	<.001
Oxygen supplementation, n (%)		222 (18.4)	160 (21.7)	62 (13.3)	<.001
High-flow nasal cannula, n (%)		65 (5.4)	55 (7.4)	10 (2.1)	<.001
Mechanical ventilator, n (%)		60 (5)	31 (4.2)	29 (6.2)	.15
Extracorporeal membrane oxygenator, n (%)		18 (1.5)	7 (0.9)	11 (2.4)	.08

### Performance of the Prediction Model

**Figure 2:**
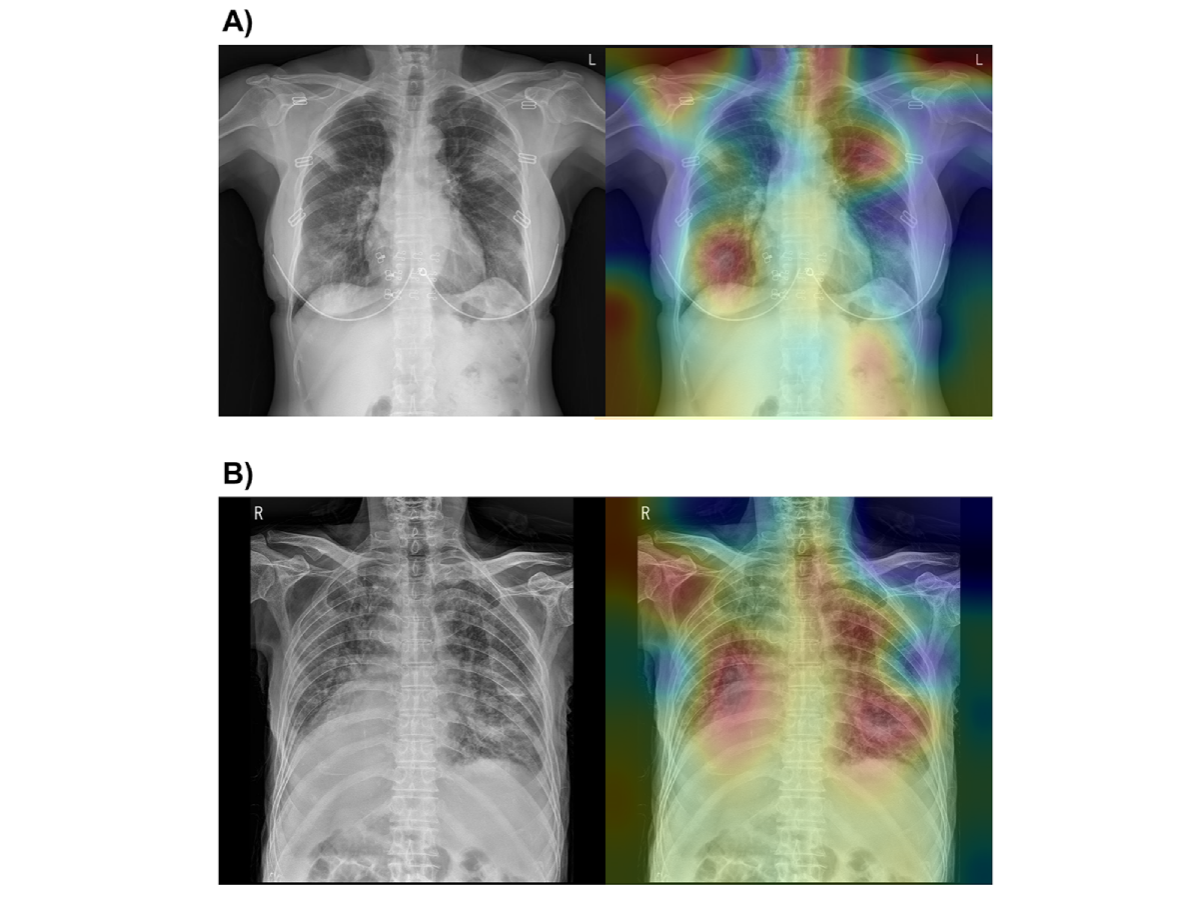
Representative cases in the test set database. (A) Chest radiograph of a 65-year-old woman who survived for 32 days after hospitalization. She had no cardiopulmonary comorbidities. She required oxygen supplementation but did not meet the operational definition of acute respiratory distress syndrome. The radiograph shows multiple consolidations and ground-glass opacities in both lung fields. The heat map mainly distinguishes the focal consolidative opacities of both lung fields. The image demonstrates red areas not only in the right lower and left upper lung fields but also around both shoulder joints, because lung segmentation was not applied in our model. The combined model, using chest radiography scores and clinical information, predicted a 40.9% chance of hospital length of stay ≤2 weeks, 74.5% chance of oxygen supplementation, and 33% chance of developing acute respiratory distress syndrome. (B) Chest radiograph of a 93-year-old man who died after 18 days of hospitalization. This patient had a previous history of heart disease. He required oxygen supplementation and met the operational definition for acute respiratory distress syndrome. The radiograph shows diffuse ground-glass opacities in both lung fields. The heat map mainly distinguishes the bilateral ground-glass opacities of both lung fields. The combined model, using chest radiography scores and clinical information, predicted a 57.8% chance of hospital length of stay ≤2 weeks, 96.4% chance of oxygen supplementation, and 99.1% chance of acute respiratory distress syndrome.

The AI model was trained, validated, and internally tested using the CXR images of the hospitalized patients with COVID-19. The performance of each model in the internal testing set is described in Multimedia Appendix 1, Figure S2. The probability of each prespecified outcome (CXR score) was calculated in the external testing set (ie, KICC-19); the AUROCs were 0.602 (95% CI 0.540-0.664) for hospital LOS ≤2 weeks, 0.647 (95% CI 0.586-0.708) for oxygen supplementation, and 0.782 (95% CI 0.720-0.845) for development of ARDS. Representative heat maps visually explain how DL preferentially recognized pneumonic lesions in the CXR images (Figure 2). The heat maps used the feature maps from the clinical data channel of the model weighted by the output probabilities.

We identified clinical variables that were significantly associated with clinical outcomes in hospitalized patients with COVID-19 (Multimedia Appendix 1, Tables S3-4; Table 3). Patients with hypertension, chronic liver disease, low lymphocyte count, or corticosteroid treatment were less likely to be discharged from the hospital within 2 weeks. Patients who needed oxygen supplementation were older; had hypertension, diabetes, or dyspnea; and had a higher level of inflammatory markers, including CRP and procalcitonin. ARDS was more common in those who were older, had dyspnea, or had higher procalcitonin levels. The performance of the prediction models using significant clinical variables was evaluated in the external testing set, with AUROCs of 0.618 (95% CI 0.558-0.678) for hospital LOS ≤2 weeks, 0.567 (95% CI 0.501-0.632) for oxygen supplementation, and 0.878 (95% CI 0.835-0.920) for development of ARDS.

**Table 3:** Univariable and multivariable logistic regression model for each clinical outcome in COVID-19 patients hospitalized at Boramae Medical Center.

Clinical outcomes			Unadjusted odds ratio (95% CI)		*P* value		Adjusted odds ratio (95% CI)		*P* value
**Hospital** **length of stay** **≤2 weeks**									
	Age	0.99 (0.98 to 1.00)		.01		1.00 (0.99 to 1.01)		.64	
	Female	1.30 (0.96 to 1.74)		.09		1.18 (0.87 to 1.61)		.29	
	Every-day smoker	0.84 (0.53 to 1.32)		.44		N/A^a^		N/A	
	Hypertension	0.57 (0.41 to 0.78)		<.001		0.68 (0.47 to 0.98)		.04	
	Diabetes mellitus	0.61 (0.41 to 0.91)		.02		0.79 (0.52 to 1.22)		.29	
	Cancer	0.91 (0.51 to 1.62)		.74		N/A		N/A	
	Cardiovascular disease	0.80 (0.45 to 1.43)		.46		N/A		N/A	
	Chronic lung disease	0.98 (0.50 to 1.91)		.95		N/A		N/A	
	Chronic kidney disease	0.28 (0.08 to 0.91)		.04		0.35 (0.11 to 1.20)		.01	
	Chronic liver disease	0.37 (0.16 to 0.86)		.02		0.40 (0.17 to 0.93)		.03	
	Cough	1.03 (0.77 to 1.39)		.84		N/A		N/A	
	Fever	1.03 (0.77 to 1.39)		.84		N/A		N/A	
	Dyspnea	1.17 (0.79 to 1.74)		.43		N/A		N/A	
	White blood cells, 1000/µL	1.04 (0.96 to 1.12)		.36		N/A		N/A	
	Lymphocyte, %	1.02 (1.01 to 1.04)		.001		1.02 (1.00 to 1.03)		.03	
	C-reactive protein >0.5 mg/dL	1.14 (0.84 to 1.53)		.40		N/A		N/A	
	Procalcitonin >0.05 ng/mL	0.82 (0.54 to 1.25)		.36		N/A		N/A	
**Oxygen supplementation**									
	Age	1.08 (1.07 to 1.10)		<.001		1.06 (1.04 to 1.08)		<.001	
	Female	0.67 (0.47 to 0.96)		.03		0.91 (0.53 to 1.56)		.73	
	Every-day smoker	0.45 (0.23 to 0.90)		.02		0.63 (0.24 to 1.69)		.36	
	Hypertension	3.85 (2.67 to 5.55)		<.001		1.90 (1.12 to 3.24)		.02	
	Diabetes mellitus	4.91 (3.23 to 7.46)		<.001		2.23 (1.24 to 4.01)		.008	
	Cancer	0.99 (0.50 to 1.98)		.98		N/A		N/A	
	Cardiovascular disease	2.86 (1.58 to 5.17)		<.001		1.24 (0.51 to 3.00)		.64	
	Chronic lung disease	2.50 (1.27 to 4.91)		.008		1.17 (0.42 to 3.27)		.77	
	Chronic kidney disease	3.18 (1.05 to 9.59)		.04		2.44 (0.48 to 12.48)		.28	
	Chronic liver disease	N/A		N/A		N/A		N/A	
	Cough	2.35 (1.61 to 3.44)		<.001		1.26 (0.74 to 2.14)		.40	
	Fever	3.13 (2.13 to 4.58)		<.001		1.55 (0.91 to 2.62)		.11	
	Dyspnea	11.04 (7.20 to 16.94)		<.001		8.93 (4.85 to 16.43)		<.001	
	White blood cells, 1000 cells/µL	1.17 (1.07 to 1.27)		<.001		0.94 (0.82 to 1.08)		.38	
	Lymphocyte, %	0.92 (0.91 to 0.94)		<.001		0.98 (0.95 to 1.01)		.11	
	C-reactive protein >0.5 mg/dL	9.52 (6.05 to 14.98)		<.001		3.04 (1.68 to 5.48)		<.001	
	Procalcitonin >0.05 ng/mL	12.01 (7.59 to 19.01)		<.001		3.50 (1.87 to 6.57)		<.001	
**Development of** **acute respiratory distress syndrome**									
	Age	1.08 (1.06 to 1.11)		<.001		1.07 (1.03 to 1.12)		<.001	
	Female	0.44 (0.25 to 0.78)		.005		0.56 (0.21 to 1.52)		.26	
	Every-day smoker	0.72 (0.28 to 1.85)		.49		N/A		N/A	
	Hypertension	4.29 (2.47 to 7.46)		<.001		1.46 (0.56 to 3.81)		.44	
	Diabetes mellitus	4.40 (2.50 to –7.75)		<.001		0.80 (0.31 to 2.10)		.65	
	Cancer	1.63 (0.66 to 3.99)		.29		N/A		N/A	
	Cardiovascular disease	3.85 (1.85 to 8.00)		<.001		1.42 (0.42 to 4.81)		.57	
	Chronic lung disease	2.34 (0.93 to 5.84)		.07		0.81 (0.20 to 3.31)		0770	
	Chronic kidney disease	2.17 (0.47 to 10.04)		.32		N/A		N/A	
	Chronic liver disease	1.07 (0.24 to 4.66)		.93		N/A		N/A	
	Cough	2.36 (1.29 to 4.33)		.006		0.52 (0.18 to 1.53)		.24	
	Fever	5.18 (2.58 to 10.41)		<.001		1.55 (0.55 to 4.40)		4.111	
	Dyspnea	18.13 (9.77 to 33.66)		<.001		12.76 (4.48 to 36.36)		<.001	
	White blood cells, 1000 cells/µL	1.28 (1.15 to 1.42)		<.001		1.04 (0.87 to 1.25)		.66	
	Lymphocyte, %	0.87 (0.84 to 0.90)		<.001		0.96 (0.91 to 1.01)		.09	
	C-reactive protein >0.5 mg/dL	14.78 (5.83 to 37.44)		<.001		2.24 (0.56 to 8.98)		.26	
	Procalcitonin >0.05 ng/mL	24.84 (13.23 to 46.63)		<.001		8.07 (2.96 to 22.00)		<.001	

^a^N/A: not applicable.

### Comparison Between Different Prediction Models

**Figure 3:**
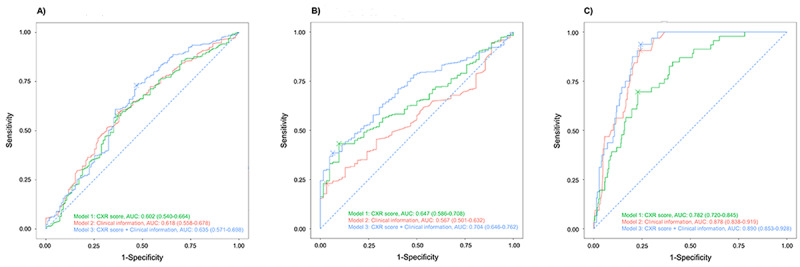
Externally validated performance of the artificial intelligence model with chest radiography score, logistic regression model with clinical information, and the combined prediction model. (A) Hospital LOS ≤2 weeks. (B) Oxygen supplementation. (C) Development of ARDS. ARDS: acute respiratory distress syndrome; AUC: area under the curve; CXR: chest radiography; LOS: length of stay.

The sensitivity, specificity, PPV, NPV, and accuracy of each model are summarized in Multimedia Appendix 1, Table S5. A comparison of the performance of the different prediction models for each clinical outcome is shown in Figure 3. We found no significant difference in the performance of the 3 prediction models in predicting hospital LOS ≤2 weeks (Multimedia Appendix 1, Figure S3). Model 3 showed an AUROC of 0.704 (95% CI 0.646-0.762) in predicting oxygen supplementation, which was significantly superior to models 1 and 2 (*P*<.001 and *P*=.02, respectively; Multimedia Appendix 1, Figure S4). Model 2 showed better performance in predicting ARDS than model 1 (*P*=.01) (Multimedia Appendix 1, Figure S5). Model 3 showed an AUROC of 0.890 (95% CI 0.853-0.928) for ARDS, which was significantly superior to model 1 (*P*=.004).

### Calibration of the Prediction Model

The calibration of models 1 and 3 in the internal and external test data sets is described in Table 4 and Multimedia Appendix 1, Figures S6 and S7. The Spiegelhalter *z* test showed good calibration of model 3 for hospital LOS ≤2 weeks, oxygen supplementation, and ARDS in the internal test set. Model 3 showed appropriate calibration only for ARDS in the external test data set (*P*=.86).

**Table 4:** Calibration of the prediction probability of the deep learning–based model using internal and external test data sets. *P* values were calculated with the Spiegelhalter *z* test; significant values indicate inappropriate calibration. Probability 1 and probability 2 were the prediction values of the deep learning–based model without and with symptom probability, respectively.

Data set and outcome				*P* value (Spiegelhalter *z* test)
**Internal test set (n=75)**				
	**Hospital length of stay ≤2 weeks**			
		Probability 1	.72	
		Probability 2	.57	
	**Oxygen supplementation**			
		Probability 1	.91	
		Probability 2	.80	
	**Development of acute respiratory distress syndrome**			
		Probability 1	.002	
		Probability 2	.81	
**External test set (n=467)**				
	**Hospital length of stay ≤2 weeks**			
		Probability 1	<.001	
		Probability 2	<.001	
	**Oxygen supplementation**			
		Probability 1	<.001	
		Probability 2	<.001	
	**Development of acute respiratory distress syndrome**			
		Probability 1	.08	
		Probability 2	.86	

## Discussion

### Principal Findings

We developed and externally validated an AI model to predict prespecified clinical outcomes based on DL using CXR. The performance of the AI model using CXR and the logistic regression model using clinical information were suboptimal for predicting hospital LOS ≤2 weeks or oxygen supplementation; there were no differences between the 2 models. The combined model, with both CXR score and clinical information, performed better in predicting oxygen supplementation. The AI prediction model for ARDS using the CXR score performed acceptably but was inferior to the model using clinical information. The combined model did not perform better in predicting ARDS than clinical information alone. CXR score calibration was appropriate for ARDS in the external test data set but not for hospital LOS ≤2 weeks or oxygen supplementation, suggesting that the CXR score may be important in identifying COVID-19 patients at high risk of progression to severe illness or ARDS. However, it is desirable to refrain from making prognoses for patients with COVID-19 based on CXR alone, considering that the predictive performance of the AI model using CXR was inferior to that of the model combining CXR score and clinical information.

CXR images in patients with COVID-19 pneumonia show various features, including diffuse ground-glass opacities, patchy reticular or nodular opacities, and consolidation [20]. The radiological features of CXR are related to the COVID-19 prognosis [21,22]. The CXR severity scoring system, which is based on radiological interpretation, is significantly associated with the prognosis of patients with COVID-19 [23]. To automate the quantification of the extent and opacity of lung lesions and the consequent assessment of radiological severity, DL models using CXR have been evaluated in COVID-19 pneumonia [24]. Recently, a DL model showed acceptable performance for predicting COVID-19 pneumonia based on CXR [12]. In our study, the CXR score derived from an AI predictive model showed superior performance for oxygen demand and comparable performance for ARDS compared to clinical information. With the application of DL techniques, CXR may need to be reconsidered as a beneficial tool for making COVID-19 pneumonia prognoses.

Hospital LOS is a clinical indicator of disease severity and time to recovery in patients with COVID-19. Prolonged hospital LOS has been associated with specific demographic characteristics and underlying comorbidities [25]. In COVID-19 patients with pneumonic infiltration in CXR, the time to negative conversion is prolonged [26]. However, none of our prediction models using CXR or clinical information were suitable for predicting whether a patient could be discharged within 2 weeks. This is consistent with a previous study reporting that radiological progression in chest CT and hospital LOS were not correlated [27]. Therefore, evidence to support an AI model using only baseline CXR to predict early recovery from COVID-19 or hospital LOS ≤2 weeks is insufficient.

Severe illness in COVID-19 is defined as a condition with SpO_2_ ≤94% in room air, including supplemental oxygen demand [28]. Progression to severe COVID-19 increases mortality risk [29]. Early prognosis and intervention may improve mortality risk in patients with COVID-19 [30,31]. Early administration of dexamethasone is associated with less progression to severe COVID-19 [32]. Therefore, many studies have attempted to predict severe COVID-19 using all available medical information, including CXR, but previous prediction models are not sufficiently validated [3]. Our prognostic model was externally validated to verify its performance in predicting oxygen supplementation need in patients with COVID-19. The AI prediction models using CXR and logistic regression with clinical information were suboptimal for predicting oxygen supplementation in patients with COVID-19. However, the predictive performance for oxygen supplementation improved to an acceptable level after clinical information was combined with the CXR score. Our results suggest that clinical and radiological information are complementary in predicting the need for oxygen supplementation.

Because of high mortality and morbidity, predicting COVID-19–associated ARDS is important [33]. CXR and clinical information, when applied to a time-dependent DL model to predict MV use, showed good predictive performance [12]. Recently, the Berlin definition of ARDS was expanded to include patients treated with HFNC [34]. Therefore, our study operationally defined ARDS as an event in which HFNC, MV, or ECMO were administered. Our AI model using CXR score showed acceptable performance; the combined model using CXR score and clinical information showed excellent performance in predicting the use of HFNC, MV, and ECMO. Further clinical trials are needed to ascertain whether early detection of patients at high ARDS risk can improve outcomes through early treatment for severe COVID-19.

### Limitations

Our study had some limitations. First, the interval between symptom onset and CXR imaging varied. The natural course of COVID-19 suggests that radiological abnormalities would have been ground-glass opacities at the beginning, progressing to consolidative lesions. Therefore, if the interval between symptom onset and CXR imaging was too short, our AI model might have underestimated the severity of prognoses among patients with COVID-19. Second, the performance of our prediction model may have changed according to vaccination history. As the vaccination rate increases, the progression to severe illness or ARDS decreases. Therefore, the PPV of our AI model may decrease in vaccinated patients. Third, most of the included patients were diagnosed with COVID-19 before results on the efficacy of dexamethasone or antiviral agents were reported [35,36]. Therefore, it is necessary to validate the performance of the AI prediction model under recently introduced standard treatments.

### Conclusions

A prediction model combining CXR score and clinical information was externally validated as having acceptable performance in predicting progression to severe illness and excellent performance in predicting the use of HFNC or MV in patients with COVID-19. We hypothesize that making prognoses with AI models using CXR could be applied for patients with COVID-19 in different settings.

## Appendix

Multimedia Appendix 1 Supplementary tables and figures.

## Abbreviations

AI: artificial intelligence
ARDS: acute respiratory distress syndrome
AUROC: area under the receiver operating characteristic curve
BMC: Boramae Medical Center
CRP: C-reactive protein
CT: computed tomography
CXR: chest radiography
DL: deep learning
ECMO: extracorporeal membrane oxygenator
HFNC: high-flow nasal cannula
KICC-19: Korean Imaging Cohort of COVID-19
LOS: length of stay
MV: mechanical ventilator
NPV: negative predictive value
PPV: positive predictive value
RCNN: region-based convolutional neural
RSNA: Radiological Society of North America
