# Correction: Deep Learning With Chest Radiographs for Making Prognoses in Patients With COVID-19: Retrospective Cohort Study

**DOI:** 10.2196/51951

**Published:** 2023-08-23

**Authors:** Hyun Woo Lee, Hyun Jun Yang, Hyungjin Kim, Ue-Hwan Kim, Dong Hyun Kim, Soon Ho Yoon, Soo-Youn Ham, Bo Da Nam, Kum Ju Chae, Dabee Lee, Jin Young Yoo, So Hyeon Bak, Jin Young Kim, Jin Hwan Kim, Ki Beom Kim, Jung Im Jung, Jae-Kwang Lim, Jong Eun Lee, Myung Jin Chung, Young Kyung Lee, Young Seon Kim, Sang Min Lee, Woocheol Kwon, Chang Min Park, Yun-Hyeon Kim, Yeon Joo Jeong, Kwang Nam Jin, Jin Mo Goo

**Affiliations:** 1 Division of Respiratory and Critical Care Department of Internal Medicine Seoul Metropolitan Government-Seoul National University Boramae Medical Center Seoul Republic of Korea; 2 College of Medicine Seoul National University Seoul Republic of Korea; 3 Department of Radiology Seoul National University Medical Research Center Seoul Republic of Korea; 4 AI Graduate School Gwangju Institute of Science and Technology Gwangju Republic of Korea; 5 Department of Radiology Seoul Metropolitan Government-Seoul National University Boramae Medical Center Seoul Republic of Korea; 6 Department of Radiology Kangbuk Samsung Hospital Sungkyunkwan University School of Medicine Seoul Republic of Korea; 7 Department of Radiology Soonchunhyang University Seoul Hospital Soonchunhyang University College of Medicine Seoul Republic of Korea; 8 Department of Radiology Research Institute of Clinical Medicine Jeonbuk National University-Biomedical Research Institute of Jeonbuk National University Hospital Jeonju Republic of Korea; 9 Department of Radiology Dankook University Hospital Cheonan Republic of Korea; 10 Department of Radiology Chungbuk National University Hospital Cheongju Republic of Korea; 11 Department of Radiology Kangwon National University Hospital Kangwon National University School of Medicine Chuncheon Republic of Korea; 12 Department of Radiology Keimyung University Dongsan Hospital Keimyung University School of Medicine Daegu Republic of Korea; 13 Department of Radiology Chungnam National University Hospital College of Medicine Daejeon Republic of Korea; 14 Department of Radiology Daegu Fatima Hospital Daegu Republic of Korea; 15 Department of Radiology Seoul St. Mary's Hospital College of Medicine, The Catholic University of Korea Seoul Republic of Korea; 16 Department of Radiology Kyungpook National University Hospital School of Medicine, Kyungpook National University Daegu Republic of Korea; 17 Department of Radiology Chonnam National University Hospital Gwangju Republic of Korea; 18 Department of Radiology Samsung Medical Center Sungkyunkwan University School of Medicine Seoul Republic of Korea; 19 Department of Radiology Seoul Medical Center Seoul Republic of Korea; 20 Department of Radiology Yeungnam University Hospital Yeungnam University College of Medicine Daegu Republic of Korea; 21 Department of Radiology and Research Institute of Radiology Asan Medical Center University of Ulsan College of Medicine Seoul Republic of Korea; 22 Department of Radiology Ewha Womans University Seoul Hospital Seoul Republic of Korea; 23 Department of Radiology Pusan National University Hospital Pusan National University School of Medicine and Biomedical Research Institute Busan Republic of Korea

In “Deep Learning With Chest Radiographs for Making Prognoses in Patients With COVID-19: Retrospective Cohort Study” (J Med Internet Res 2023;25:e42717) the authors made one amendment. The affiliation for co-author Soo-Youn Ham was listed as:

Department of Radiology, Kangbuk Samsung Hospital, Seoul, Korea, Republic of

And will be changed to read as follows:

Department of Radiology, Kangbuk Samsung Hospital, Sungkyunkwan University School of Medicine, Seoul, Korea, Republic of

The correction will appear in the online version of the paper on the JMIR Publications website on August 23, 2023 together with the publication of this correction notice. Because this was made after submission to PubMed, PubMed Central, and other full-text repositories, the corrected article has also been resubmitted to those repositories.

