# Supplementary material for: Deep Learning With Chest Radiographs for Making Prognoses in Patients With COVID-19: Retrospective Cohort Study
Source: J Med Internet Res. 2023 Feb 16;25:e42717. doi: 10.2196/42717 (PMC9937110; doi:10.2196/42717)
Supplement: Multimedia Appendix 1 [file jmir_v25i1e42717_app1.docx]

**Supplementary Table 1. Baseline characteristics of the patients diagnosed with COVID-19 in different datasets**

|  | **Train set (n=589)** | **Validation set (n=75)** | **Internal testing set (n=75)** | **p-value** |
| --- | --- | --- | --- | --- |
| **Age, mean (SD)** | 54.6 (17.3) | 54.9 (18.0) | 52.6 (19.3) | 0.627 |
| **Female (%)** | 296 (50.3) | 43 (57.3) | 37 (49.3) | 0.493 |
| **BMI, mean (SD)** | 24.0 (4.1) | 23.4 (3.4) | 24.0 (3.7) | 0.460 |
| **Ever-smoker, n (%)** | 64 (10.9) | 11 (14.7) | 5 (6.7) | 0.437 |
| **Comorbidities** |  |  |  |  |
| Hypertension, n (%) | 172 (29.2) | 21 (28.0) | 20 (26.7) | 0.889 |
| Diabetes mellitus, n (%) | 87 (14.8) | 15 (20.0) | 16 (21.3) | 0.207 |
| Chronic lung disease, n (%) | 27 (4.6) | 5 (6.7) | 6 (8.0) | 0.370 |
| Chronic kidney disease, n (%) | 10 (1.7) | 1 (1.3) | 2 (2.7) | 0.799 |
| Chronic liver disease, n (%) | 20 (3.4) | 3 (4.0) | 1 (1.3) | 0.591 |
| Cerebrovascular disease, n (%) | 38 (6.5) | 3 (4.0) | 1 (1.3) | 0.158 |
| Cardiovascular disease n (%) | 37 (6.3) | 5 (6.7) | 8 (10.7) | 0.363 |
| Cancer, n (%) | 43 (7.3) | 5 (6.7) | 3 (4.0) | 0.567 |

BMI, body mass index; SD, standard deviation

**Supplementary Table 2. Clinical manifestations of the patients diagnosed with COVID-19 in different datasets**

|  | **Train set (n=589)** | **Validation set (n=75)** | **Internal testing set (n=75)** | **p-value** |
| --- | --- | --- | --- | --- |
| **Symptoms** |  |  |  |  |
| Abnormality in sense of smell and taste, n (%) | 110 (18.7) | 16 (21.3) | 16 (21.3) | 0.762 |
| Myalgia, n (%) | 184 (31.2) | 22 (29.3) | 16 (21.3) | 0.209 |
| Sore throat, n (%) | 173 (29.4) | 24 (32.0) | 17 (22.7) | 0.401 |
| Cough, n (%) | 330 (56.0) | 50 (66.7) | 37 (49.3) | 0.092 |
| Sputum, n (%) | 248 (42.1) | 33 (44.0) | 32 (42.7) | 0.951 |
| Chest discomfort, n (%) | 96 (16.3) | 8 (10.7) | 11 (14.7) | 0.437 |
| Dyspnea , n (%) | 99 (16.8) | 14 (18.7) | 15 (20.0) | 0.753 |
| Fever, n (%) | 297 (50.4) | 38 (50.7) | 41 (54.7) | 0.786 |
| Rhinorrhea or nasal obstruction, n (%) | 81 (13.8) | 13 (17.3) | 9 (12.0) | 0.615 |
| Diarrhea, n (%) | 99 (16.8) | 14 (18.7) | 16 (21.3) | 0.597 |
| **Laboratory tests** |  |  |  |  |
| WBC, mean (SD) | 5,032 (2,000) | 4,691 (1,522) | 4,896 (1,739) | 0.325 |
| Lymphocyte %, mean (SD) | 29.1 (11.3) | 31.3 (11.8) | 30.7 (11.7) | 0.192 |
| hs-CRP, median (IQR) | 0.67 (0.18–3.27) | 0.42 (0.12–1.79) | 0.62 (0.23–2.93) | 0.439 |
| Procalcitonin, median (IQR) | 0.03 (0.02–0.05) | 0.03 (0.02–0.04) | 0.03 (0.02­–0.05) | 0.820 |
| Troponin I, median (IQR) | 4.30 (2.70–7.60) | 3.40 (2.40–7.05) | 5.05 (3.20–9.95) | 0.751 |
| LDH, median (IQR) | 214 (182–270) | 212 (180–251) | 214 (183–251) | 0.753 |
| **Treatment** |  |  |  |  |
| Hydroxychloroquine, n (%) | 15 (2.5) | 1 (1.3) | 2 (2.7) | 0.806 |
| Remdesivir, n (%) | 42 (7.1) | 5 (6.7) | 11 (14.7) | 0.068 |
| Antibiotics, n (%) | 165 (28.0) | 22 (29.3) | 24 (32.0) | 0.762 |
| Corticosteroid, n (%) | 56 (9.5) | 5 (6.7) | 13 (17.3) | 0.062 |
| **Length of stay, median (IQR)** | 13 (10–19) | 13 (10–18.5) | 14 (10–20.5) | 0.345 |
| **Length of stay ≤2 weeks (%)** | 361 (61.3) | 48 (64.0) | 42 (56.0) | 0.579 |
| **Oxygen supplementation, n (%)** | 121 (20.5) | 15 (20.0) | 24 (32.0) | 0.071 |
| **HFNC, n (%)** | 38 (6.5) | 7 (9.3) | 10 (13.3) | 0.082 |
| **MV, n (%)** | 21 (3.6) | 3 (4.0) | 7 (9.3) | 0.064 |
| **ECMO, n (%)** | 4 (0.7) | 1 (1.3) | 2 (2.7) | 0.231 |

ECMO, extracorporeal membrane oxygenator; HFNC, high-flow nasal cannula; hs-CRP, high-sensitivity C-reactive protein; IQR, interquartile range; LDH, lactate dehydrogenase; MV, mechanical ventilator; SD, standard deviation; WBC, white blood cell

**Supplementary Table 3. Baseline characteristics of the patients diagnosed with COVID-19 according to different outcomes**

|  | **Hospital LOS ≤2 weeks** | | | **Oxygen supplementation** | | | **Development of ARDS** | | |
| --- | --- | --- | --- | --- | --- | --- | --- | --- | --- |
|  | **Yes, n=451** | **No, n=288** | **P-value** | **Yes, n=160** | **No, n=579** | **P-value** | **Yes, n=58** | **No, n=681** | **P-value** |
| **Age, mean (SD)** | 53.1 (16.7) | 56.6 (18.8) | 0.008 | 68.4 (12.7) | 50.6 (16.8) | <0.001 | 71.2 (12.5) | 53.0 (17.3) | <0.001 |
| **Female (%)** | 241 ( 53.4) | 135 (46.9) | 0.096 | 69 ( 43.1) | 307 (53.0) | 0.033 | 19 (32.8) | 357 (52.4) | 0.006 |
| **BMI, mean (SD)** | 24.0 (3.6) | 23.9 (4.6) | 0.746 | 24.5 (3.8) | 23.8 (4.1) | 0.057 | 24.2 (3.9) | 23.9 (4.1) | 0.646 |
| **Current smoker, n (%)** | 48 (10.6) | 36 (12.5) | 0.511 | 10 (6.2) | 74 (12.8) | 0.031 | 5 (8.6) | 79 (11.6) | 0.638 |
| **Comorbidities** |  |  |  |  |  |  |  |  |  |
| Hypertension, n (%) | 109 (24.2) | 104 (36.1) | 0.001 | 84 ( 52.5) | 129 (22.3) | <0.001 | 35 (60.3) | 178 (26.1) | <0.001 |
| Diabetes mellitus, n (%) | 60 (13.3) | 58 (20.1) | 0.018 | 58 ( 36.2) | 60 (10.4) | <0.001 | 24 (41.4) | 94 (13.8) | <0.001 |
| Chronic lung disease, n (%) | 23 (5.1) | 15 ( 5.2) | 1.000 | 15 (9.4) | 23 ( 4.0) | 0.011 | 6 (10.3) | 32 (4.7) | 0.119 |
| Chronic kidney disease, n (%) | 4 (0.9) | 9 ( 3.1) | 0.049 | 6 (3.8) | 7 ( 1.2) | 0.068 | 2 (3.4) | 11 (1.6) | 0.618 |
| Chronic liver disease, n (%) | 9 (2.0) | 15 ( 5.2) | 0.029 | 8 (5.0) | 16 ( 2.8) | 0.246 | 2 (3.4) | 22 (3.2) | 1.000 |
| Cerebrovascular disease, n (%) | 21 (4.7) | 21 ( 7.3) | 0.178 | 25 ( 15.6) | 17 ( 2.9) | <0.001 | 7 (12.1) | 35 (5.1) | 0.058 |
| Cardiovascular disease n (%) | 28 (6.2) | 22 ( 7.6) | 0.545 | 21 ( 13.1) | 29 ( 5.0) | 0.001 | 11 (19.0) | 39 (5.7) | <0.001 |
| Cancer, n (%) | 30 (6.7) | 21 ( 7.3) | 0.853 | 11 (6.9) | 40 ( 6.9) | 1.000 | 6 (10.3) | 45 (6.6) | 0.419 |

BMI, body mass index; SD, standard deviation

**Supplementary Table 4. Clinical manifestations of the patients diagnosed with COVID-19 according to different outcomes**

|  | **Hospital LOS ≤2 weeks** | | | **Oxygen supplementation** | | | **Development of ARDS** | | |
| --- | --- | --- | --- | --- | --- | --- | --- | --- | --- |
|  | **Yes, n=451** | **No, n=288** | **P-value** | **Yes, n=160** | **No, n=579** | **P-value** | **Yes, n=58** | **No, n=681** | **P-value** |
| **Symptoms** |  |  |  |  |  |  |  |  |  |
| Abnormality in sense of smell and taste, n (%) | 107 (23.7) | 35 (12.2) | <0.001 | 18 (11.2) | 124 (21.4) | 0.006 | 7 (12.1) | 135 (19.8) | 0.206 |
| Myalgia, n (%) | 144 (31.9) | 78 (27.1) | 0.187 | 46 (28.7) | 176 (30.4) | 0.760 | 15 (25.9) | 207 (30.4) | 0.566 |
| Sore throat, n (%) | 146 (32.4) | 68 (23.6) | 0.013 | 30 (18.8) | 184 (31.8) | 0.002 | 9 (15.5) | 205 (30.1) | 0.028 |
| Cough, n (%) | 256 (56.8) | 161 (55.9) | 0.878 | 115 (71.9) | 302 (52.2) | <0.001 | 43 (74.1) | 374 (54.9) | 0.007 |
| Sputum, n (%) | 195 (43.2) | 118 (41.0) | 0.595 | 94 (58.8) | 219 (37.8) | <0.001 | 44 (75.9) | 269 (39.5) | <0.001 |
| Chest discomfort, n (%) | 74 (16.4) | 41 (14.2) | 0.490 | 42 (26.2) | 73 (12.6) | <0.001 | 17 (29.3) | 98 (14.4) | 0.005 |
| Dyspnea , n (%) | 82 (18.2) | 46 (16.0) | 0.492 | 80 (50.0) | 48 (8.3) | <0.001 | 42 (72.4) | 86 (12.6) | <0.001 |
| Fever, n (%) | 231 (51.2) | 145 (50.3) | 0.876 | 115 (71.9) | 261 (45.1) | <0.001 | 48 (82.8) | 328 (48.2) | <0.001 |
| Rhinorrhea or nasal obstruction, n (%) | 65 (14.4) | 38 (13.2) | 0.721 | 14 (8.8) | 89 (15.4) | 0.044 | 8 (13.8) | 95 (14.0) | 1.000 |
| Diarrhea, n (%) | 77 (17.1) | 52 (18.1) | 0.807 | 42 (26.2) | 87 (15.0) | 0.001 | 20 (34.5) | 109 (16.0) | 0.001 |
| **Laboratory tests** |  |  |  |  |  |  |  |  |  |
| WBC, mean (SD) | 5,035 (1,933) | 4,904 (1,930) | 0.368 | 5491(2725) | 4844 (1622) | <0.001 | 6200 (3688) | 4880 (1666) | <0.001 |
| Lymphocyte %, mean (SD) | 30.9 (11.7) | 27.8 (10.7) | 0.001 | 22.5 (11.3) | 31.5 (10.7) | <0.001 | 17.0 (10.1) | 30.6 (10.9) | <0.001 |
| CRP, median (IQR) | 0.42 (0.15–1.53) | 0.42 (0.35–0.51) | 0.3 | 1.44 (0.42–5.52) | 0.42 (0.25–0.48) | <0.001 | 2.80 (0.42–7.60) | 0.42 (0.25–0.69) | <0.001 |
| Procalcitonin, median (IQR) | 0.03 (0.02–0.03) | 0.03 (0.02–0.03) | 0.244 | 0.03 (0.03–0.07) | 0.03 (0.02–0.03) | <0.001 | 0.06 (0.03–0.13) | 0.03 (0.02–0.03) | <0.001 |
| Troponin I, median (IQR) | 4.20 (2.70­–6.50) | 4.70 (2.60–9.55) | 0.192 | 7.90 (4.5–14.65) | 3.60 (2.50–5.85) | <0.001 | 13.6 (7.8–20.0) | 3.9 (2.6–6.5) | <0.001 |
| LDH, median (IQR) | 219 (182–270) | 210 (180–253) | 0.306 | 296 (221–369) | 204 (177–242) | <0.001 | 369 (277–486) | 210 (179–262) | <0.001 |
| **Treatment** |  |  |  |  |  |  |  |  |  |
| Hydroxychloroquine, n (%) | 2 (0.4) | 16 (5.6) | <0.001 | 2 (1.2) | 16 (2.8) | 0.418 | 0 (0.0) | 18 (2.6) | 0.418 |
| Remdesivir, n (%) | 22 (4.9) | 36 (12.5) | <0.001 | 58 (36.2) | 0 (0.0) | <0.001 | 33 (56.9) | 25 (3.7) | <0.001 |
| Antibiotics, n (%) | 86 ( 19.1) | 125 (43.4) | <0.001 | 130 (81.2) | 81 (14.0) | <0.001 | 57 (98.3) | 154 (22.6) | <0.001 |
| Corticosteroid, n (%) | 25 (5.5) | 49 (17.0) | <0.001 | 74 (46.2) | 0 (0.0) | <0.001 | 36 (62.1) | 38 (5.6) | <0.001 |

ECMO, extracorporeal membrane oxygenator; HFNC, high-flow nasal cannula; hs-CRP, high-sensitivity C-reactive protein; IQR, interquartile range; LDH, lactate dehydrogenase; MV, mechanical ventilator; SD, standard deviation; WBC, white blood cell

**Supplementary Table 5. Predictive performances for different clinical outcomes**

|  | Hospital LOS ≤2 weeks | | | Oxygen supplementation | | | Development of ARDS | | | |
| --- | --- | --- | --- | --- | --- | --- | --- | --- | --- | --- |
|  | **Model 1** | **Model 2** | **Model 3** | **Model 1** | **Model 2** | **Model 3** | **Model 1** | **Model 2** | **Model 3** |  |
| Sensitivity | 0.573 (0.520­0.625) | 0.638 (0.585-0.688) | 0.740 (0.691-0.785) | 0.432 (0.383-0.492) | 0.230 (0.190-0.274) | 0.358 (0.338-0.435) | 0.761 (0.512-0.874) | 0.875 (0.710-0.965) | 0.969 (0.838-0.999) |  |
| Specificity | 0.630 (0.534­0.720) | 0.595 (0.497-0.687) | 0.523 (0.425-0.619) | 0.903 (0.801-0.964) | 0.968 (0.888-0.996) | 0.935 (0.843-0.982) | 0.781 (0.739-0.820) | 0.776 (0.733-0.815) | 0.712 (0.666-0.755) |  |
| Positive predictive value | 0.833 (0.780-0.877) | 0.834 (0.785-0.877) | 0.834 (0.789-0.874) | 0.967 (0.929-0.988) | 0.979 (0.926-0.997) | 0.975 (0.937-0.993) | 0.276 (0.200-0.362) | 0.230 (0.158-0.314) | 0.204 (0.143-0.277) |  |
| Negative predictive value | 0.315 (0.255-0.381) | 0.338 (0.272-0.410) | 0.382 (0.304-0.466) | 0.196 (0.151-0.247) | 0.161 (0.125-0.203) | 0.189 (0.147-0.237) | 0.968 (0.943-0.984) | 0.988 (0.969-0.997) | 0.997 (0.982-1.000) |  |
| Accuracy | 0.586 (0.541-0.632) | 0.627 (0.582-0.671) | 0.688 (0.645-0.731) | 0.495 (0.448-0.541) | 0.328 (0.285-0.372) | 0.458 (0.412-0.505) | 0.779 (0.739-0.816) | 0.783 (0.742-0.820) | 0.730 (0.687-0.770) |  |

Three different prediction models were evaluated for each clinical outcome: Model 1 using the CXR score derived from the deep learning model, Model 2 using clinical information derived from a multivariable regression model, and Model 3 using both the CXR score and clinical information derived from the multivariable regression model.

**Supplementary Figure 1.** Illustration of faster region-based convolutional neural networks architecture

**
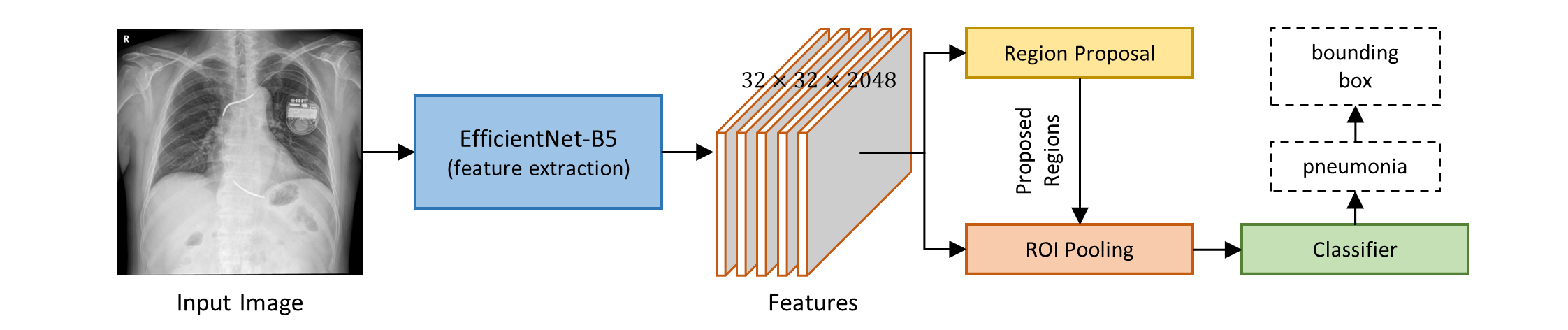
**

**Supplementary Figure 2.** Performance of the artificial intelligence model with a CXR score, logistic regression model with clinical information, and combined prediction model in the internal testing set

**
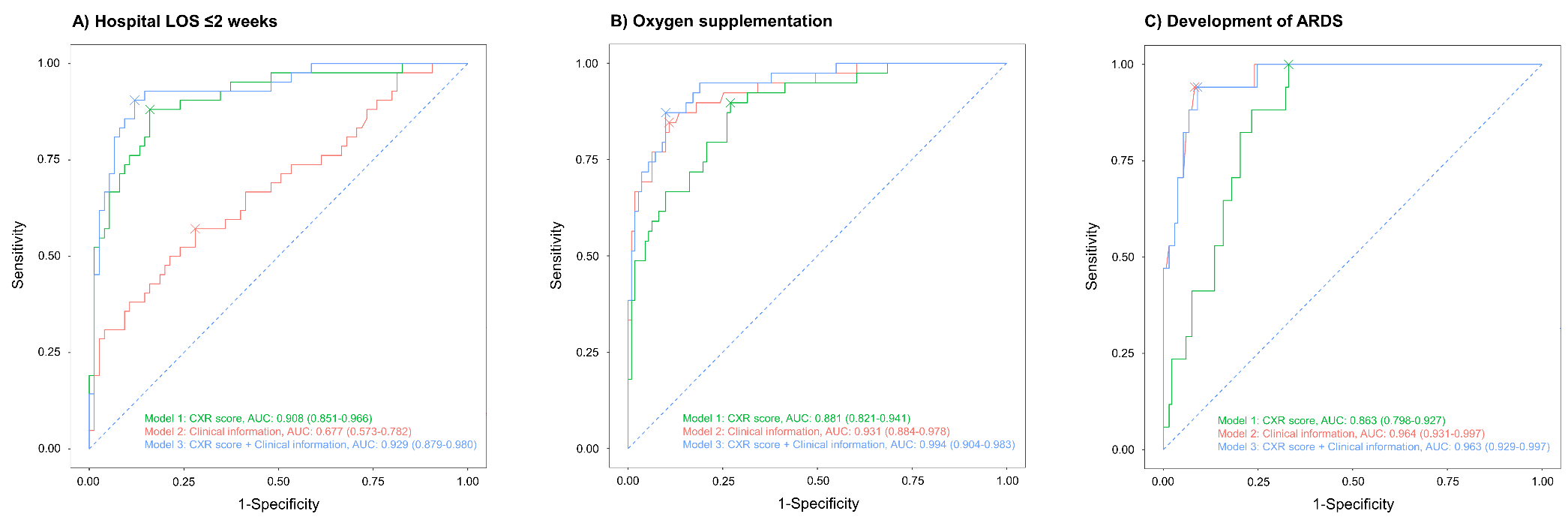
**

Model 1, artificial intelligence model with CXR score; Model 2, logistic regression model with clinical information; Model 3, combined prediction model

A) Model 1 vs. Model 2, P-value<0.001; Model 1 vs. Model 3, P-value<0.001; Model 2 vs. Model 3, P-value=0.119

B) Model 1 vs. Model 2, P-value=0.139; Model 1 vs. Model 3, P-value=0.028; Model 2 vs. Model 3, P-value=0.123

C) Model 1 vs. Model 2, P-value=0.001; Model 1 vs. Model 3, P-value=0.001; Model 2 vs. Model 3, P-value=0.465

**Supplementary Figure 3.** Performance of the prediction models for hospital length of stay ≤2 weeks


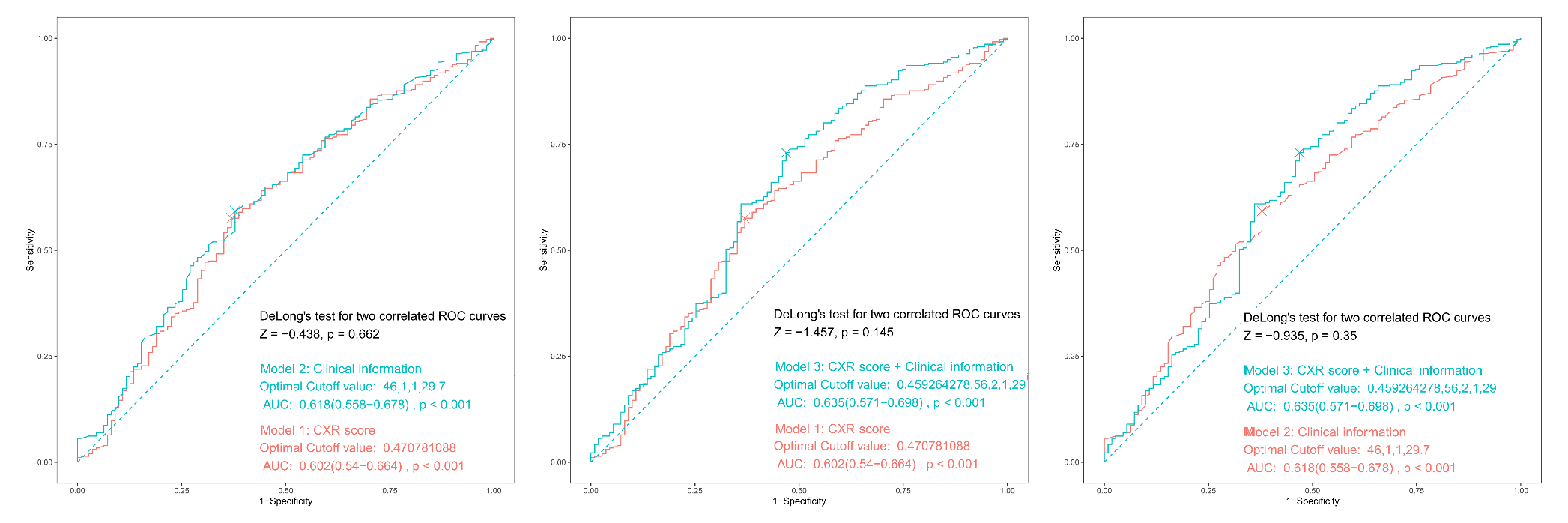


Model 1, artificial intelligence model with CXR score; Model 2, logistic regression model with clinical information; Model 3, combined prediction model

**Supplementary Figure 4.** Performance of the prediction models for oxygen supplementation

**
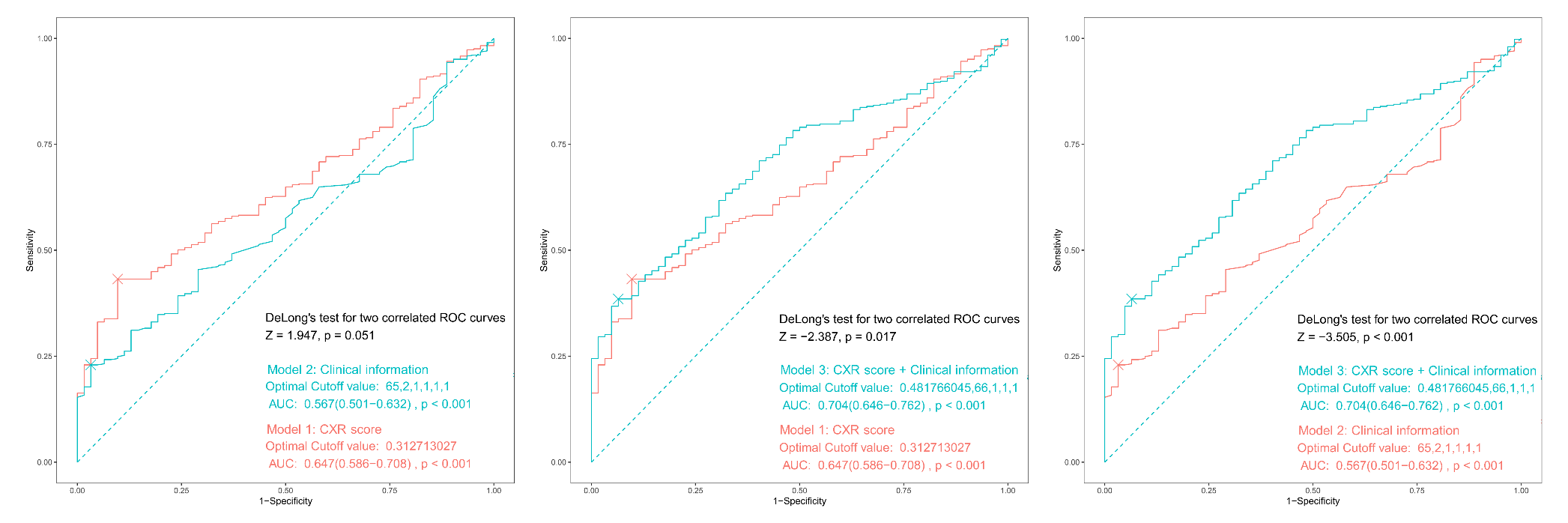
**

Model 1, artificial intelligence model with CXR score; Model 2, logistic regression model with clinical information; Model 3, combined prediction model

**Supplementary Figure 5.** Performance of the prediction models for acute respiratory distress syndrome

**
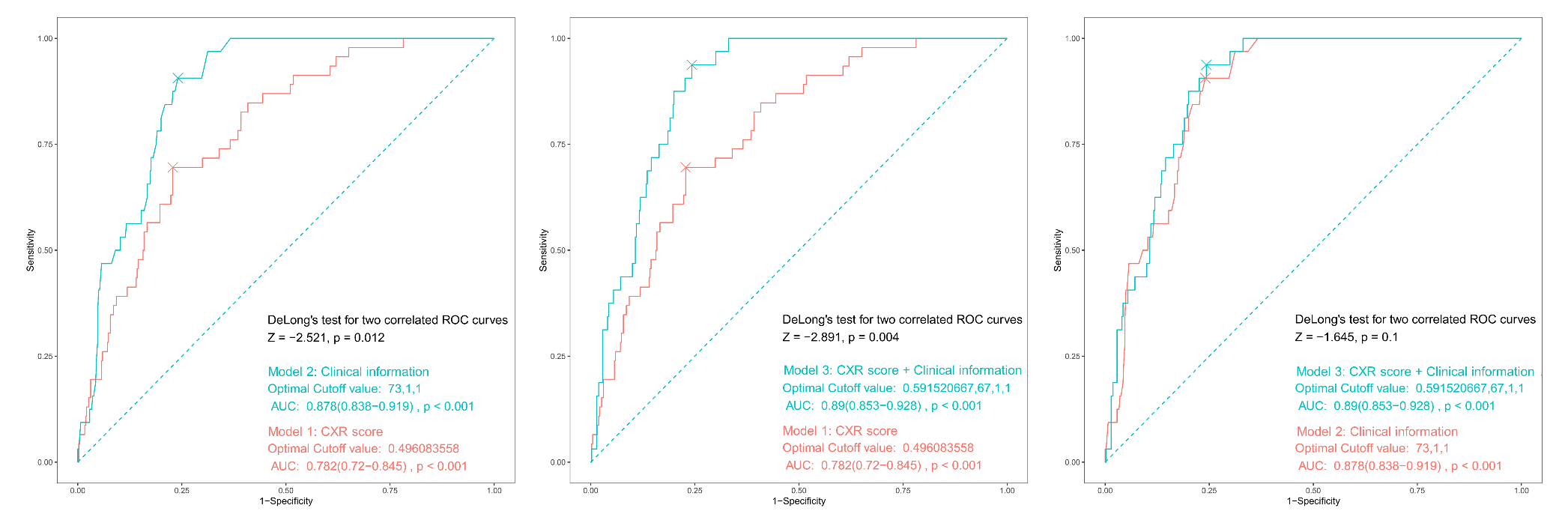
**

Model 1, artificial intelligence model with CXR score; Model 2, logistic regression model with clinical information; Model 3, combined prediction model

**Supplementary Figure 6.** Calibration plots of the predictive probability of the deep learning-based model using the internal test dataset

**
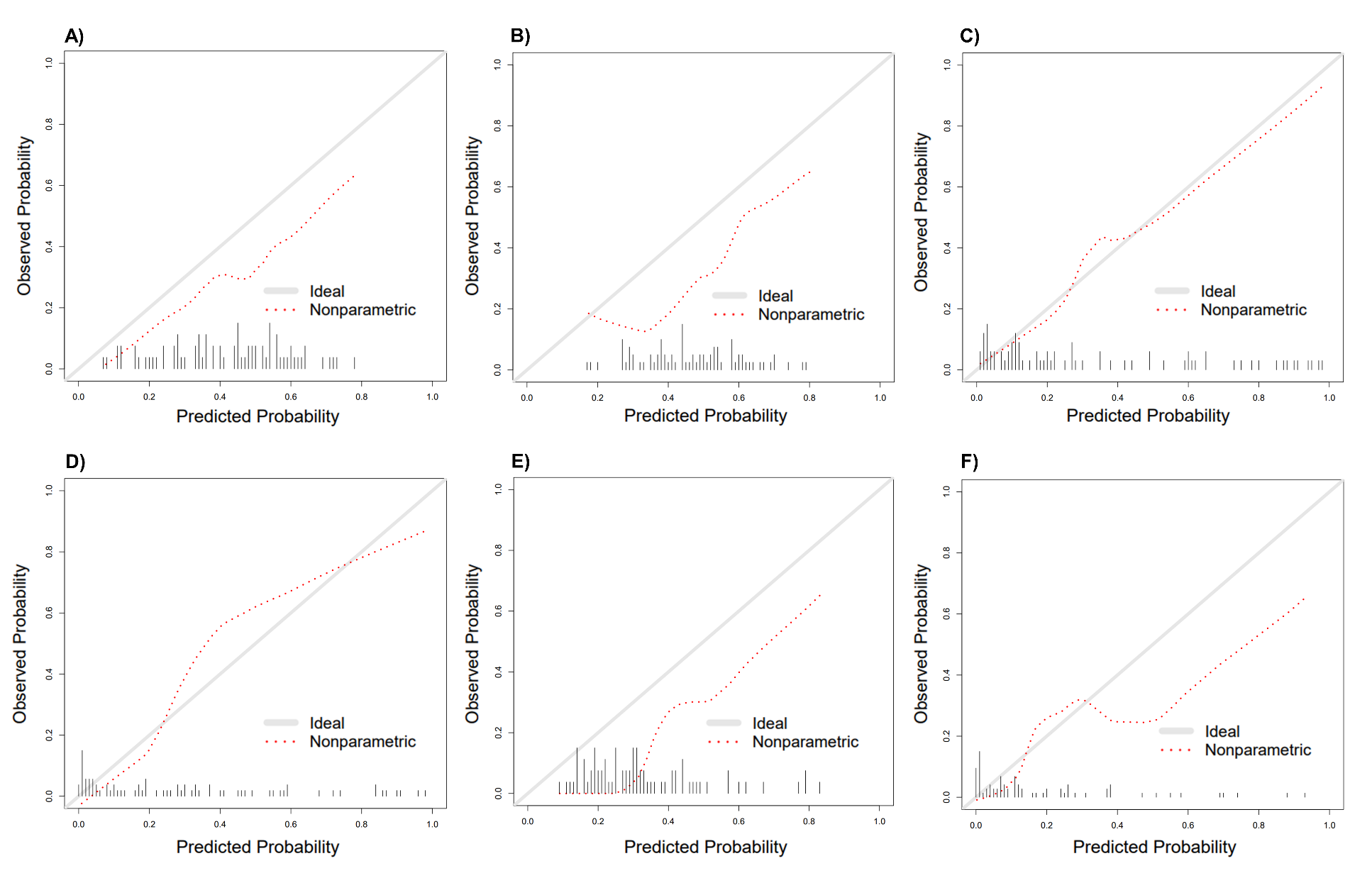
**

Model 1, artificial intelligence model with CXR score; Model 2, logistic regression model with clinical information; Model 3, combined prediction model

A) Calibration curve of the predictive probability of Model 1 for hospital length of stay ≤2 weeks in the internal test set (n=75)

B) Calibration curve of the predictive probability of Model 3 for hospital length of stay ≤2 weeks in the internal test set (n=75)

C) Calibration curve of the predictive probability of Model 1 for oxygen supplementation in the internal test set (n=75)

D) Calibration curve of the predictive probability of Model 3 for oxygen supplementation in the internal test set (n=75)

E) Calibration curve of the predictive probability of Model 1 for development of acute respiratory distress syndrome in the internal test set (n=75)

F) Calibration curve of the predictive probability of Model 3 for development of acute respiratory distress syndrome in the internal test set (n=75)

**Supplementary Figure 7.** Calibration plots of the predictive probability of the deep learning-based model using the external test dataset

**
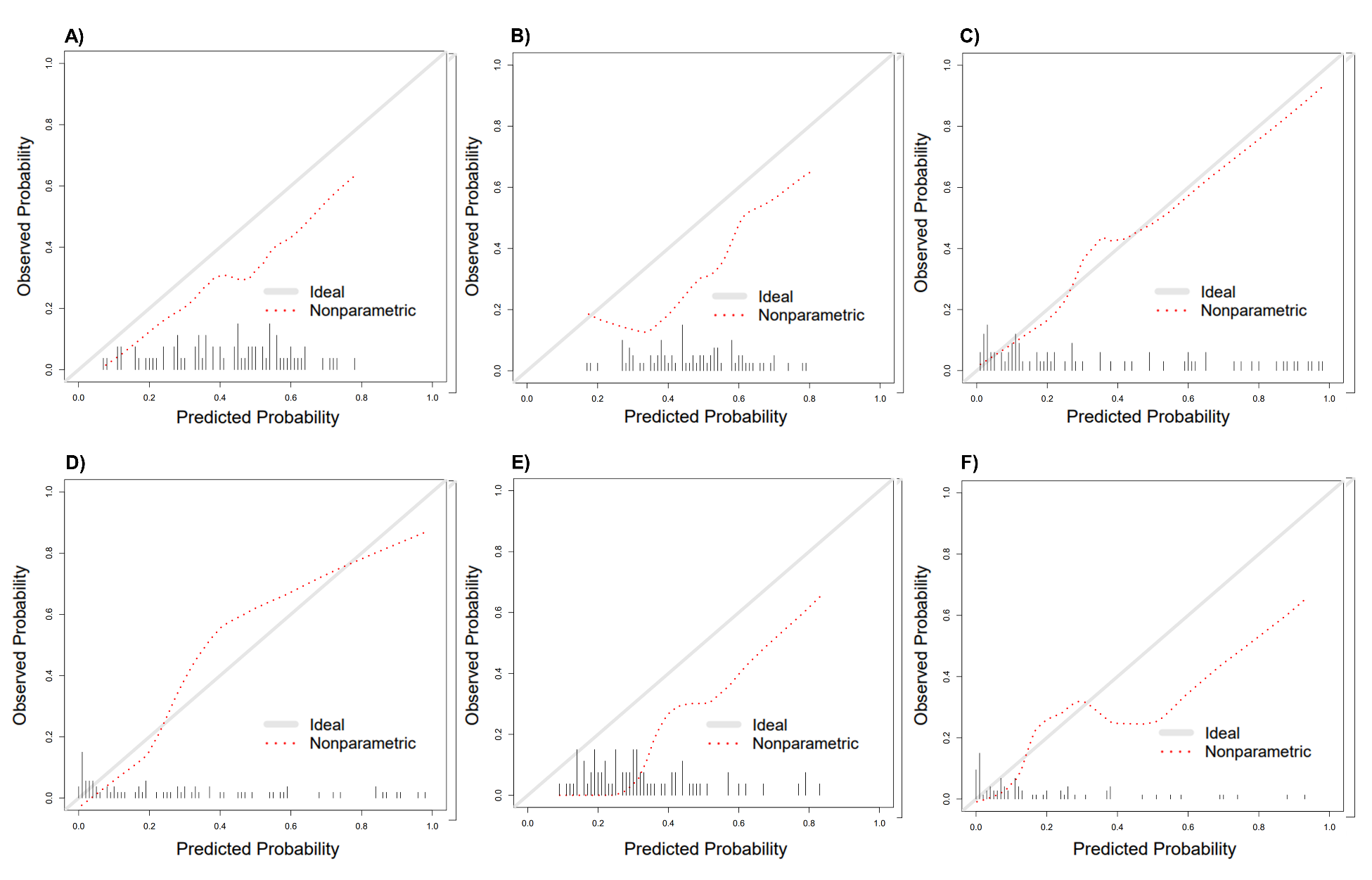
**

Model 1, artificial intelligence model with CXR score; Model 2, logistic regression model with clinical information; Model 3, combined prediction model

A) Calibration curve of the predictive probability of Model 1 for hospital length of stay ≤2 weeks in the external test set (n=467)

B) Calibration curve of the predictive probability of Model 3 for hospital length of stay ≤2 weeks in the external test set (n=467)

C) Calibration curve of the predictive probability of Model 1 for oxygen supplementation in the external test set (n=467)

D) Calibration curve of the predictive probability of Model 3 for oxygen supplementation in the external test set (n=467)

E) Calibration curve of the predictive probability of Model 1 for the development of acute respiratory distress syndrome in the external test set (n=467)

F) Calibration curve of the predictive probability of Model 3 for the development of acute respiratory distress syndrome in the external test set (n=467)
